# Evaluation of an Indirect Immunofluorescence Assay for the Detection of *Anaplasma phagocytophilum* Antigen in Ovine Buffy Coat Smears

**DOI:** 10.3390/microorganisms10020276

**Published:** 2022-01-25

**Authors:** Labrini V. Athanasiou, Constantina N. Tsokana, Eleni G. Katsogiannou, Sofia Boutsini, Panagiotis D. Katsoulos

**Affiliations:** 1 Department of Medicine, Faculty of Veterinary Medicine, University of Thessaly, 43100 Karditsa, Greece; lathan@vet.uth.gr (L.V.A.); kotsokan@vet.uth.gr (C.N.T.); elkatsog@uth.gr (E.G.K.); 2 Parasitology–Parasitic Diseases, Entomology and Bee Health Department, Veterinary Centre of Athens, General Directorate of Veterinary Services, 15341 Athens, Greece; sboutsini@yahoo.gr; 3 Clinic of Farm Animals, Faculty of Health Sciences, School of Veterinary Medicine, Aristotle University of Thessaloniki, 54627 Thessaloniki, Greece

**Keywords:** anaplasmosis, buffy coat, cytology, immunofluorescence assay for antigen detection (Ag-IFAT), sheep

## Abstract

Diagnosis of anaplasmosis is challenging considering the great variation in clinical signs and the limitations of the available diagnostic assays, while the detection of carrier animals that play a significant role in disease epidemiology as reservoirs is of great significance. In this study, we evaluated the diagnostic accuracy of a newly developed indirect immunofluorescent assay (Ag-IFAT) for the detection of *A. phagocytophilum* antigens in buffy coat specimens, alone and in combination with cytology, using PCR as a reference. Blood samples were collected from 138 sheep of the Chios breed from six farms in Greece. A buffy coat was extruded from the centrifuged blood. Buffy coat smears were used for cytological examination and the Ag-IFAT assay. The Ag-IFAT assay presented excellent specificity (100%) and high sensitivity (85.4%) for the detection of *A. phagocytophilum* antigens in buffy coats, and it has an almost perfect agreement with PCR and cytology (κ value = 0.88 and 0.85, respectively). *A. phagocytophilum* antigens are likely to be detected using Ag-IFAT in a PCR-positive animal, as indicated by the good performance of the assay. Overall, this assay presents high diagnostic accuracy, and it could be used for the detection of animals during the early stage of infection.

## 1. Introduction

Members of the genus *Anaplasma* are obligate intracellular alpha-protobacteria, coccoid to ellipsoidal, often pleomorphic, gram-negative, non-motile, and measuring 0.4 to 1.3 or 2 μm in size. They reside and replicate in membrane-bound vacuoles within the cytoplasm of eukaryotic host cells [1]. After staining with Romanowsky stain, they appear as purple-colored mulberry-like microcolonies called “morulae” with diameters measuring 1.5 to 2.5 or 6 μm [2,3,4]. The genus comprises six species: *Anaplasma marginale*, *A. centrale*, *A. ovis*, *A. phagocytophilum*, *A. bovis* and *A. platys*. Members of the genus *Anaplasma* present differences in their cellular tropism, geographical distribution, host range, vectors and pathogenicity [1].

*A. phagocytophilum* is of remarkable significance in both human and veterinary medicine. Its wide host range includes humans, carnivores, ruminants, rodents, insectivores, birds and reptiles [5]. It is largely distributed across Europe, the USA, and Asia. Transmission of *A. phagocytophilum* involves ticks belonging to the *Ixodes* genus [6,7]. Although *A. phagocytophilum* DNA has been detected in other tick species, their vector competence and their role in the epidemiology of anaplasmosis are still unclear [1]. Vertebrate hosts develop persistent infections and act as a source of infection for ticks [8]. Following transmission to a vertebrate host, *A. phagocytophilum* infects neutrophils, eosinophils, lymphocytes and monocytes, leading to the development of leukopenia, neutropenia and reduction in neutrophil function, with subsequent immunosuppression that may promote the occurrence of opportunistic infections. The phase of bacteremia, the host susceptibility and the bacterial strain involved highly affect the percentage of phagocytic cells that will be infected [7]. 

In humans, *A. phagocytophilum* causes human granulocytic anaplasmosis (HGA) and it is the causative agent of tick-borne fever (TBF) in sheep and goats and pasture fever in cattle [6]. TBF is a challenging and wasting condition with severe economic impact and welfare challenges in the small ruminant industry [9]. High fever (>41 °C) is the most characteristic symptom of the disease in domestic ruminants, while weakness and anorexia are also commonly reported. Although TBF is seldom fatal, an increased incidence of secondary infections, such as tick pyemia (caused by *Staphylococcus* spp.) or *Mannheimia septicaemia*, is reported due to immunosuppression. Abortion, stillbirth, reduced milk yield, impaired spermatogenesis in males, low fertility in sheep and reduced weight gain in young animals are among the most commonly occurring complications [2,6,9,10]. 

The contribution of clinical signs in diagnosis is rather limited. Microscopic examination of peripheral blood-stained smears, as well as serological and molecular assays, are commonly used for the diagnosis of anaplasmosis. At the onset of the infection, blue intracytoplasmic inclusion microcolonies can usually be observed in granulocytes. On the contrary, diagnosis through blood smear cytological examination may not be feasible to identify carrier animals. Thus, serological assays, such as indirect immunofluorescence antibody (Ab-IFAT), enzyme-linked immunosorbent assay (ELISA) and complement fixation tests (CF), have been used for the detection of anti-*Anaplasma* antibodies [11,12,13,14,15]. Among the various serological techniques, competitive ELISA (cELISA) presents high sensitivity and specificity for the detection of *Anaplasma*-specific antibodies [16], while the Ab-IFAT has been commonly used in epidemiological studies. However, due to the time needed to elapse from infection to antibody appearance in peripheral blood serology is of limited value soon after infection [12,17]. PCR presents several advantages, including its high sensitivity even in the early stages of infection, when the *Anaplasma* load in blood cells is low [17], the differentiation between subspecies, and the detection of coinfections with multiple *Anaplasma* subspecies [17,18,19].

The collection of blood samples during the early acute phase of symptoms is crucial for all direct tests, including microscopic examination and molecular assays. During this stage of infection, sufficient numbers of bacteria are present in the circulating blood, making their detection more probable. The leukocyte tropism of *A. phagocytophilum,* together with the very few infected leukocytes circulating as a result of leukopenia, make buffy coat a preferred sample compared to whole blood [18]. 

The diagnosis of anaplasmosis is challenging considering the great variation in clinical signs and the limitations of the available diagnostic assays, while the detection of carrier animals that play an important role in disease epidemiology as reservoirs is of great significance. Furthermore, the substantial economic impact associated with livestock infection and the zoonotic potential of *A. phagocytophilum* makes the employment of accurate direct laboratory tests a necessity. In this study, we aimed to evaluate the diagnostic accuracy of a newly developed indirect immunofluorescent assay for the detection of *A. phagocytophilum* antigens (Ag-IFAT) in buffy coat specimens, alone and in combination with cytology in buffy coat smears, using an established PCR assay as a reference.

## 2. Materials and Methods

### 2.1. Animals

Blood samples collected from a total number of 138 sheep of the Chios breed from six farms in Greece were used in the study. Details on sampling have been previously published [20].

### 2.2. Inclusion and Exclusion Criteria 

The inclusion and exclusion criteria have been previously published [20] and comprised of (1) the presence of ticks, (2) the absence of other ectoparasites, such as fleas and lice, (3) deworming at least 2 months before their selection in the study, and (4) no cytologic or serologic evidence of concurrent tickborne infections (*Borrelia burgdorferi*, *Babesia* sp. and *Theileria* sp.).

### 2.3. Allocation of Animals in Groups

Allocation of the animals in Groups A–D (A: sheep with the presence of the *A. phagocytophilum* inclusions in buffy coat smear, group B: sheep being positive in cytology and serology, group C: sheep with antibodies against *A. phagocytophilum,* and group D: sheep found negative in cytology and serology) has been previously published [20]. 

#### 2.3.1. Cytological Examination

For cytological examination of the buffy coat, smears were air-dried, fixed with methanol and Giemsa stained. Up to 1000 oil immersion fields were screened for *A. phagocytophilum* in each of the Giemsa-stained smears, as previously described [20,21].

#### 2.3.2. Immunofluorescence Assays

An immunofluorescence assay for the detection of antibodies against *A. phagocytophilum* (Ab-IFAT) has been previously published [20].

For immunofluorescence assay for the detection of *A. phagocytophilum* antigen (Ag-IFAT) to be performed, a buffy coat smear was air dried, then fixed in 95% ethanol for 10 min, air dried again, and placed at −20 °C pending analysis. After removing the slides from the freezer, a rectangle was defined by etching a line with a diamond tipped etching pencil ventricularly to the long axis of the slide at the direction of the feathered edge of the smear. Then, the slides were again placed in 95% ethanol for 30 sec and rinsed in deionized water for 5 min. Serum of horses, positive for antibodies against *A. phagocytophilum,* was placed on the etched area of the slide (MegaFLUO^®^ ANAPLASMA *phagocytophilum*, Megacor Veterinary Diagnostics, Hörbranz, Austria). The slides were then placed in a suitable chamber created by placing a moist paper towel in a standard Petri dish and incubating at 37 °C for 30 min. After incubation, the samples were washed several times with phosphate-buffered saline (PBS). Then, the defined area of the slides was covered by anti-horse FITC IgG conjugate (MegaFLUO^®^ ANAPLASMA *phagocytophilum*, Megacor Veterinary Diagnostics, Hörbranz, Austria). After incubation at 37 °C for 30 min, the samples were washed several times with phosphate-buffered saline (PBS). Mounting fluid was added and the samples were read with an oil immersion objective lens (100×) on a Nikon Eclipse E-400 fluorescent microscope (Nikon, Badhoevedorp, The Netherlands). 

#### 2.3.3. DNA Extraction and PCR Assay

Total genomic DNA extraction from whole blood samples in EDTA was performed using a commercially available DNA extraction kit (Nucleo Spin Blood Quick Pure kit, Macherey-Nagel, Dueren, Germany) according to the manufacturer’s instructions. The extracted DNA was stored at −20 °C pending analysis. 

DNA extracts were examined for the presence of *A. phagocytophilum* DNA with a species-specific PCR targeting a 122 bp fragment of the msp2 gene, using the primers 903f (5′-AGTTTGACTGGAACACACCTGATC-3′) and 1024r (5′-CTCGTAACCAATCTCAAGCTCAAC-3′) [22,23]. A final PCR mix volume of 25 μL was prepared by adding 15 pmol of each primer, 12.5 μL of Taq DNA Promega GoTaq^®^ Hot Start Colorless Master Mix (Promega Corporation, Madison, WI, USA), 7 μL DNA-free water and 2.5 μL of extracted DNA. Amplification was undertaken in a thermocycler (Applied Biosystems^®^ Veriti^®^ Thermal Cycler, Applied Biosystems, Waltham, MA, USA) with the following cycling conditions: initial denaturation at 95 °C for 5 min, followed by 35 cycles of denaturation at 94 °C for 20 sec, annealing at 50 °C for 30 sec, and extension at 72 °C for one min, and a final extension at 72 °C for 10 min, as previously described [23]. Positive and negative controls were used in each PCR run. PCR-grade water was used as a negative control. Amplification products were subjected to electrophoresis in 2% agarose gel stained with ethidium bromide (0.5 μg/mL) and visualized under ultraviolet light. 

### 2.4. Statistical Analysis

The agreement among the results of the tests performed for the detection of the *A. phagocytophilum* antigen was measured using the Cohen’s Kappa (κ) value. A value of 0 indicates poor agreement, while a value of 1 indicates perfect agreement [24,25]. For the assessment of the diagnostic performance of the tests (cytology, Ag-IFAT and their combination, in series or in parallel), the calculation of the sensitivity, specificity, positive likelihood ratio (PLR) and negative likelihood ratio (NLR) was performed, using MedCalc Statistical Software version 14.8.1 (MedCalc Software bvba, Ostend, Belgium; http://www.medcalc.org; 2014). PLR values > 10 and NLR values < 0.1 are indicative of good test performance [26].

## 3. Results

Overall, 33/138 (23.91%) samples were positive for the detection of *A. phagocytophilum* in buffy coat, 41/138 (29.71%) samples for the detection of antigens by the Ag-IFAT and 48/138 (34.78%) samples were positive in PCR. 

Following the allocation of the samples in Groups A–D as previously described [20], the positive samples in Ag-IFAT, cytology and PCR that were found in this study and the positive samples in Ab-IFAT are presented in Table 1. More samples in the early infection (Group D, serology and cytology negative samples) and seropositive stage could be detected with Ag-IFAT compared to cytology, close enough to the number of samples that could be detected with PCR. 

Sensitivity, specificity, PLR and NLR values for cytological examination of buffy coat smears, Ag-IFAT and their combination (in series and in parallel) against PCR, which was considered as the reference method, are presented in Table 2. Ag-IFAT presented higher sensitivity compared to cytology (85.42% and 68.75%, respectively) and the same sensitivity to that observed for the combination of Ag-IFAT and cytology in parallel. Both methods and their combination in series or in parallel presented excellent specificity (100%). The PLR values could not be calculated, as the denominator of the equation was zero due to the 100% specificity, suggesting a good test performance for both methods and their combination. The NLR values were better (lower) for Ag-IFAT and the combination of Ag-IFAT and cytology in parallel (0.15 in both cases). 

As shown in Table 3, an almost perfect agreement was observed between Ag-IFAT and cytological examination of buffy coat smears (κ value = 0.85) or Ag-IFAT and PCR in blood samples (κ value = 0.88). On the other hand, cytological examination and PCR presented substantial agreement (κ value = 0.74). 

## 4. Discussion

In this study, we evaluated the diagnostic accuracy of a newly developed indirect immunofluorescent assay for the detection of *A. phagocytophilum* antigens (Ag-IFAT) in buffy coat specimens, alone and in combination with cytology in buffy coat smears, using an established PCR assay as a reference.

The diagnostic accuracy of Ag-IFAT was high compared to PCR, suggesting that it could be a good candidate method for *A. phagocytophilum* antigen detection even during the early infection stage in sheep. In particular, Ag-IFAT presented high sensitivity (85.42%) and excellent specificity (100%) for the detection of *A. phagocytophilum* infection in sheep. The two methods presented an almost perfect agreement (κ value = 0.88) and based on the PLR value, which was indicative of good performance, *A. phagocytophilum* antigens are likely to be detected using Ag-IFAT for a PCR-positive animal. The best (lowest) NLR value was also observed for Ag-IFAT. However, it was indicative of moderate power to identify PCR-negative samples when antigens were not detected in Ag-IFAT.

Several high-performance PCR tests have been developed for the detection of *A. phagocytophilum* DNA in blood and tissue samples, including conventional, nested and real-time targeting of 16S rRNA, msp4, groEL, ankA and p44 genes [27,28]. PCR assays present high sensitivity, provide a reliable diagnosis even in the early stages of infection, enable the identification of different *Anaplasma* spp. and the detection of mixed infections [18,29]. Whole blood and buffy coat are considered the preferred samples for molecular screening due to the cell tropism of *Anaplasma* spp. [17,18,30]. In a previous study, PCR presented 100% sensitivity compared to microscopic examination, cELISA and Ab-IFAT. In that study, a lower percentage of animals were found to be blood smear positive or seropositive, suggesting that PCR positive results can be obtained even during the early stage of infection when *Anaplasma* infected cells are low or antibodies have not been produced respectively [17].

Regarding blood smear examination, its diagnostic accuracy may be affected by several parameters, such as the low number of infected cells, the levels of bacteremia, the degree of neutropenia, monocytopenia, thrombocytopenia, anemia and the occurrence of intracellular artifacts, Döhle and Howell–Jolly bodies or other inclusions, and it highly depends on the experience of the examiner [17,18]. Thus, although it is the quickest and most convenient laboratory test that is traditionally used for the diagnosis of clinical anaplasmosis, it presents the lowest sensitivity compared to other laboratory methods. In fact, its accuracy increases in recently acquired infections, except in cases of severe anemia. For ruminants, the examination of 400 granulocytes is considered sufficient to detect infected leucocytes in recent diseases [31,32]. On the other hand, its use is of limited value for the detection of pre-symptomatic or persistently infected animals due to the low numbers of circulating *Anaplasma*-infected cells. An experimental study in sheep showed that *Anaplasma* inclusions could be observed continuously for 2 weeks starting from day 3 post-infection, although in some animals they were detected sporadically up to day 52 post-infection [33]. Thus, a negative cytological result does not rule out infection, and other laboratory tests should be performed if persistent infections are suspected [17,18]. In this study, an almost perfect correlation was found between Ag-IFAT and cytology in buffy coat smears (κ value = 0.85). The sensitivity observed was higher for Ag-IFAT than for cytology and for the combination of the two methods in series (68.75%). However, similarly high sensitivity was observed for the combination of Ag-IFAT and cytology in parallel (85.42%), suggesting that cytology in buffy coat does not increase the sensitivity of Ag-IFAT alone. In this study, the buffy coat was chosen as the preferred sample for cytology in order to increase the possibility of detecting *A. phagocytophilum* inclusions compared to whole blood smears. As has been previously shown, a lower sensitivity is expected in whole blood compared to buffy coat smears [21]. However, the usefulness of whole blood cytological examination is not limited to the detection of *A. phagocytophilum* inclusions in blood cells. As previously shown, whole blood smear examination can also provide valuable information on the cell count and morphology for monitoring disease progression in sheep. Leucopenia in combination with thrombocytopenia raises a strong suspicion of anaplasmosis, while the reduction of blood cell counts along with positivity in cytological examination and serology are also observed in the acute phase of the disease [20].

Likewise, serologic assays are of limited value in the early acute phase of infection due to the absence of detectable antibodies. Experimental studies showed that the detection of anti-*Anaplasma* antibodies occurs mainly on day 7 after infection or day 5 in some cases, reaching the highest antibody titers on day 14 [34,35,36]. The competitive ELISA that is currently used for diagnosis of anaplasmosis recognizes the MSP5 antigen in *A. marginale, A. central*, *A. ovis* and *A. phagocytophilum,* which is conserved among all known *Anaplasma* spp. [37]. This is another limitation of serological techniques; cross-reactivity among the different *Anaplasma* spp. has been reported for cELISA and IFAT, as well as the CF test [38,39], making the identification of the species involved impossible without the use of molecular assays. As for *A. phagocytophilum*, cross-reaction with *A. marginale* has been previously reported with IFAT, and it has been suggested that this is also possible with *A. ovis* [39]. Although data on the prevalence and clinical importance of *A. ovis* in sheep in Greece is lacking, cross-reaction with Ag-IFAT cannot be ruled out. Recently, higher sensitivity and specificity of cELISA (91.9% sensitivity and 86.9% specificity) has been reported compared to cytological examination of blood smears (62.2% vs. 43.6%, respectively) for caprine and ovine anaplasmosis [17]. Concerning Ab-IFAT, several studies support its use for the diagnosis of *Anaplasma* spp. in sheep [13,40,41]. Recently, Ab-IFAT was shown to have high sensitivity (100%) and specificity (91.9%) when compared to the combination of microscopic examination and cELISA, while a previous study reported a similar level of specificity and sensitivity for Ab-IFAT when compared with cELISA [15,17].

**Figure 1:**
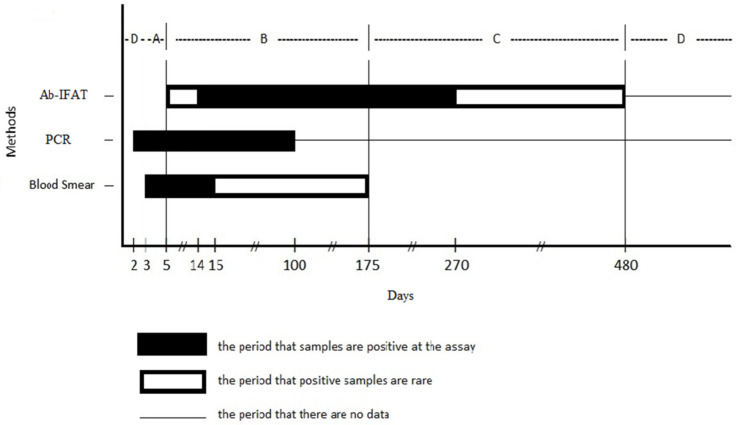
The period when antibodies against *A. phagocytophilum* are detected using Ab-IFAT, the period when DNA of *A. phagocytophilum* is detected in blood (PCR) and the period when *A. phagocytophilum* in blood smear is detected.

In a previous study, the evolution of antibodies after experimental infection and the detection of *A. phagocytophilum* in the blood smear in the course of infection was graphically presented [20]. In that figure, the most probable corresponding position of each group (Groups A–D) was annotated. This graph was further modified in this study in order to include the PCR positive results (Figure 1).

More specifically, based on previous studies, PCR becomes positive 2 days after experimental infection and remains positive for up to 100 days [42,43]. This information is depicted in Figure 1 and corresponds to sheep of Groups A, B and D. Regarding group D, where sheep were found negative in cytology and serology, it had been assumed that either the sheep had never been infected before, excluding an infection in the past with full recovery, or they were in a very early stage of infection. This assumption is further supported by the PCR results of this study, as five PCR-positive animals were detected in Group D (serology and cytology negative) showing an early infection stage. Concerning the newly developed Ag-IFAT assay, although less sensitive than PCR, it seems to position the animals in a similar infection stage. Moreover, compared to PCR, Ag-IFAT is of lower cost and less time-consuming. Cytology is by far the most affordable method. It presents, however, lower sensitivity compared to Ag-IFAT. Finally, compared to Ab-IFAT, the new assay requires the same time to be performed but is less expensive.

## 5. Conclusions

The newly developed Ag-IFAT assay presents excellent specificity and high sensitivity when PCR is used as the reference method for the detection of *A. phagocytophilum* antigens in buffy coat and it has almost perfect agreement with PCR and cytology. *A. phagocytophilum* antigens are likely to be detected using Ag-IFAT in a PCR-positive animal, as indicated by the good performance of the assay. Overall, this assay presents a high diagnostic accuracy, and it could be used for the detection of animals during the early stage of infection.

## Figures and Tables

**Table 1:** The number of samples that were found positive in cytology, Ag-IFAT and PCR in each group.

	Groups (*N*)			
Method	A (17)	B (16)	C (16)	D (89)
Cytology	17	16	0	0
Ab-IFAT	0	16	16	0
Ag-IFAT	17	16	6	2
PCR	17	16	10	5

Group A: sheep with the presence of the *A. phagocytophilum* inclusions in buffy coat smear, group B: sheep being positive in cytology and serology, group C: sheep with antibodies against *A. phagocytophilum*, and group D: sheep found negative in cytology and serology. *N*: number of samples; Ab-IFAT: indirect immunofluorescent assay for antibody detection; Ag-IFAT: indirect immunofluorescent assay for antigen detection; PCR: polymerase chain reaction.

**Table 2:** Sensitivity, specificity, positive likelihood ratio and negative likelihood ratio of cytology, indirect immunofluorescent assay for antigen detection (Ag-IFAT) and their combination, in series and in parallel, for the detection of *A. phagocytophilum* in ovine blood samples.

	Cytology	Ag-IFAT	Cytology and Ag-IFAT, in Series	Cytology or Ag-IFAT, in Parallel
Sens%	68.75	85.42	68.75	85.42
95% CI	53.75–81.34	72.24–93.93	53.75–81.34	72.24–93.93
Spec%	100	100	100	100
95% CI	95.98–100	95.98–100	95.98–100	95.98–100
PLR	-	-	-	-
95% CI	-	-	-	-
NLR	0.31	0.15	0.31	0.15
95% CI	0.21–0.48	0.07–0.29	0.21–0.48	0.07–0.29

Ag-IFAT: indirect immunofluorescent assay for antigen detection; Sens: Sensitivity; Spec: Specificity; PLR: positive likelihood ratio; NLR: negative likelihood ratio; CI: confidence interval.

**Table 3:** Agreement among cytology, indirect immunofluorescence assay for antigen detection (Ag-IFAT) and PCR for the detection of *A. phagocytophilum* in ovine blood samples.

Method	κ Value	95% CI
Cytology vs. PCR	0.742	0.622–0.861
Ag-IFAT vs. PCR	0.884	0.801–0.967
Cytology vs. FA	0.853	0.755–0.951

Ag-IFAT: indirect immunofluorescent assay for antigen detection; PCR: polymerase chain reaction.

## Data Availability

The data presented in this study are available on request from the corresponding author. The data are not publicly available due to further processing for other studies.
